# Arson—Mental Alienation

**Published:** 1848-07-01

**Authors:** 


### ARSON.—MENTAL ALIENATION.

A LEGAL investigation recently took place in the district of Lavaux, Switzerland, respecting a fire in the village of Forel, which occurred on the 4th of August, 1847, and destroyed an inn, which had been purchased a few days previously by George Louis L——, from Pierre V——. Suspicious circumstances having attended the occurrence, in consequence of a quarrel having arisen between the vendor and the purchaser prior to the fire, the *juge de paix* caused inquiries to be instituted; Madame R——, an inhabitant of Forel, called on him during the investigation, and acknowledged herself guilty of the crime, which she alleged she had committed in order to be revenged of L——, jun., by whom she believed herself to be pregnant, and who had recently treated her with great unkindness. Remorse, and the fear of innocent persons being punished for her crime, led her to acknowledge her guilt. She was immediately sent to prison, and while there was visited by Dr. Fonjallaz and Dr. Rogiveu, who in their official report, stated that they could not discover any traces of insanity; and that she expressed herself clearly and lucidly on all subjects on which they conversed, but was invariably silent respecting the alleged case of arson.

When brought to trial in February last, she was described as being forty-eight years old, but appearing older than she was, of tall stature, and thin, with a sombre and melancholy expression of countenance. She answered questions at first slowly, and frequently not until they had been repeated several times, but afterwards replied more readily and more in detail. She again acknowledged having set the house on fire, but varied her tale so far as to allege that Pierre V——, the vendor, had urged her to do it; had supplied the matches, and was present at the commission of the crime, and further, that she had not any cause of quarrel with the purchaser or his son.

It appeared from the examination of one witness that her character was bad, having been stained with several acts of libertinage, while another person stated that she had once attempted to commit suicide by throwing herself into a well. This latter charge she explained by stating that she had something the matter with her hand, and that she merely sat upon the edge of the well, and dipped her hand in.

The court directed that the prisoner should be examined by Drs. Recordon, Perey, and Zimmer, relative to the state of her mind, and she was accordingly transferred to the hospital for the insane at Lausanne. The report, which was presented to the court on the 19th of April, was of exceeding length, but completely established the insanity of the accused. It examined first the early history, then the acts bearing on the crime of which she was accused, and finally her conduct while in the asylum. Madame R—— was stated to be of the melancholic temperament, tall and thin, her features expressive of sadness, and also she had arrived at a critical age. Her character during childhood was singular, unquiet, and troublesome. She was afterwards tormented with the fear of dying from hunger, although not in a position to render such a dread feasible. While in the service of the Prefect of Levaux, she exhibited such unequivocal signs of insanity that she was dismissed, although she had for years previously done her work well, and did not give cause of complaint. Her hallucinations appeared to consist in hearing guns fired, and the belief that armed men were coming to take her into custody; she was very irrascible and quarrelsome with her companions, and passed her nights without sleeping—the latter being mentioned as a very characteristic trait of insanity. With respect to the alleged commission of the crime, the reporters consider that the mode in which she stated she had effected it, was in itself an evidence of insanity; she left home early, fired the house, returned without presenting any indications of her guilt, and then calls upon the judge, and proffered her self-accusation. The absence of any reasonable, or at least apparent motive for the crime, is dwelt upon as having a great influence on the diagnosis. Madame R—— had not any cause of quarrel with L——, the purchaser, or his family; and the alleged connexion with L——'s son, and the pregnancy, they do not regard as a sufficient motive, as she was still living with her husband. With respect to her complicity with V——, the motive is still less evident, for she states that V—— neither gave her, nor promised her anything, to induce her to become an incendiary. The reporters were further of opinion that she did not present any indications of pyromania, because in such a case, the general characteristics of monomania are discernible, which was not the case with her.

Her conduct while in the asylum offered irrefragable proofs of insanity. Like the insane generally, she did not testify any surprise at finding herself there; she appeared constantly plunged in stupor, and scarcely ever replied to the questions put to her. When she did, she would interrupt a sensible remark that might fall from her by a question which was either absurd, or had not any reference to the subject of the conversation. One while she appeared very fond of one woman, loaded her with caresses, and called her her sister; a few days afterwards, another female became the object of her affection. When secretly watched, her conduct appeared to be just the same; if sitting, she was continually balancing her body, automatically—a common practice with the insane. Soon after her admission, she heard cries and voices proceeding from the walls of her cell, but would never tell what the voices said to her. She also manifested a certain degree of insensibility to cold. The sleep was disturbed and restless, and the pulse varied greatly; generally very slow, it sometimes became remarkably quick. Finally, although she manifested indifference to the crime with which she was charged, and its consequences, she on three occasions attempted to destroy herself by strangulation.

The reporters consequently concluded that Madame R—— was affected with true mental alienation, with hallucinations, and that it presented the characters of cype mania, with a tendency to suicide. In their subsequent examination before the court, they expressed themselves unanimously in this opinion, and M. Percy observed that it was not a rare occurrence for the insane to accuse themselves of imaginary crimes, or of those which they had not committed. Some years ago, he said, a child was found in one of the cantons of Switzerland, which had evidently been murdered. A girl accused herself of being both the mother and the murderess of the deceased, and she related very circumstantially the details of the crime; nevertheless, after a very long process, it was ascertained that she had never been pregnant.

The counsel for the prosecution, while admitting the insanity and consequent non-responsibility of Madame R——, expressed his opinion that she was guilty of firing the house, and called upon the jury to return a verdict accordingly. On the other hand, it was alleged that she had not committed the crime, and that her confession was in itself a proof of her insanity. The verdict of the jury negatived the charge, and Madame R—— was consequently set at liberty.—*Gazette des Tribunaux.*

